# High-Density Lipoproteins and Cardiovascular Disease: The Good, the Bad, and the Future II

**DOI:** 10.3390/biomedicines10030620

**Published:** 2022-03-07

**Authors:** Josep Julve, Joan Carles Escolà-Gil

**Affiliations:** 1 Institut d’Investigacions Biomèdiques IIB Sant Pau, 08041 Barcelona, Spain; 2 CIBER de Diabetes y Enfermedades Metabólicas Asociadas (CIBERDEM), 28029 Madrid, Spain; 3 Departament de Bioquímica i Biologia Molecular, Universitat Autònoma de Barcelona, 08041 Barcelona, Spain

The notion that high-density lipoproteins (HDL) are atheroprotective is supported by different lines of evidence. Although early epidemiological studies suggested that HDL cholesterol (HDL-C) concentrations are inversely related to cardiovascular disease (CVD), data from recent clinical trials showed that simply raising HDL-C failed to confer protection against future cardiovascular events. Such studies raised the hypothesis that functional atheroprotective properties of HDL rather than their circulating concentrations should be eventually targeted in HDL-based therapies. Thus, strategies aimed at improving HDL function rather than simply increasing plasma HDL-C concentrations should be considered. In this Special Issue of *Biomedicines* titled “High-Density Lipoproteins and Cardiovascular Disease: The Good, the Bad, and the Future II”, these relevant matters are debated in nine articles that were contributed by global experts in the field.

The notion that the atheroprotective properties of HDL are strongly determined by HDL function was addressed by Lorkowski and Smith [1]. These authors described several cell-based and cell-free assays that are commonly used in different clinical studies to evaluate the potential of HDL against coronary heart disease [1]. In this regard, Bonizzi et al. further described the clinical significance of the intrinsic quantitative and qualitative characteristics of HDL in different cardiometabolic stress conditions, i.e., obesity, type 2 diabetes mellitus, and CVD [2]. Notably, the authors also rescued the potential of HDL-C in combination with other biomarkers related to systemic inflammation, i.e., elevations in the monocyte cell count, as a candidate biomarker for the diagnosis and/or prognosis of CVD. Furthermore, an increased monocyte cell count/HDL-C ratio has recently been reported in subjects with chronic kidney disease (CKD), which is commonly associated with an increased CVD [3]. Remarkably, this ratio predicts major cardiovascular events during follow-up in CKD subjects.

HDL transports a cargo comprising multiple proteins and lipid species with several bioactive properties that favourably influence several biological processes. Lappegård et al. emphasized the importance of evaluating these atheroprotective functions in HDL subfractions and their causal relationship with clinical disease [4]. In particular, the application of methodologies such as Lipoprint^®^ has enabled the subfractionation of serum lipoproteins in different clinical settings. In this context, Coimbra S et al. elegantly reviewed the usefulness of this approach to assess HDL subpopulations in subjects with CKD and their functional implications [5]. HDL metabolism and remodeling are frequently altered in CKD pathologies [6,7,8]. In line with this, the HDL size has been reported to be predominantly larger in CKD subjects [9], being the latter linked to altered lipid and protein moieties and impaired HDL-mediated cholesterol efflux capacity. These findings are in line with the paradoxical coexistence of larger HDL and increased risk for adverse CVD outcomes in the context of CKD. Supporting to this, recent evidence also suggests a positive causal relationship between HDL size and the risk of myocardial infarction [10]. Overall, these findings reinforce the message that increasing the number of small HDL particles, which are involved in the removal of excess cholesterol, would provide protection against future CVD events. In this regard, functional studies have recently shown that smaller HDL species promote both ATP-binding cassette transporter (ABC) A1-dependent cholesterol efflux and anti-inflammatory effects in whole blood [11].

HDL dysfunction is one of the main features of FH patients, thereby contributing to their high CVD risk. This concept was developed in another review article of this Special Issue [12] describing that an altered HDL metabolism and function are intrinsic features of familial hypercholesterolemia (FH). Although HDL particles constitute the first defensive barrier against the burden of high low-density lipoprotein (LDL) cholesterol levels, whether HDL function can be ameliorated by the current standard disease treatments needs to be further investigated.

Several reports have demonstrated the prognostic value of both HDL-mediated macrophage cholesterol efflux and the gut microbial-derived metabolite trimethylamine N-oxide (TMAO) in predicting cardiovascular mortality in patients with myocardial infarction [13,14]. A report published in this Special Issue demonstrated that a reduced ABCA1/G1-mediated macrophage cholesterol efflux was independently associated with mortality in patients with ST-segment elevation myocardial infarction. Although the circulating concentrations of TMAO were higher in the deceased patients, this change did not remain significantly associated with mortality after statistical adjustment. Furthermore, neither TMAO nor their precursors affected macrophage cholesterol efflux to HDL or their association with mortality [15].

Another report of this Special Issue applied shotgun proteomics to identify the protein signatures in both HDL and LDL from healthy volunteers and atherosclerotic patients who had undergone carotid endarterectomy. In total, 84 and 16 proteins were found to be differentially expressed in HDL and LDL. These findings widen the HDL and LDL proteome with two and twenty-one additional proteins involved in the inflammatory and immune and coagulation pathways and allow protein signatures to be identified specifically for patients with “hard” or “soft” plaques [16].

The use of oxidized HDL (oxHDL) as a candidate biomarker for CVD was also assessed in another review published in this Special Issue [17]. Remarkably, oxHDL has been reported to accumulate in atherosclerotic plaques [18], and it was highlighted the clinical significance of circulating levels of oxHDL in the context of CVD [17]. In this regard, the serum concentrations of oxHDL have been concomitantly associated with cardiometabolic diseases [17]. Although the serum elevations of oxHDL are not completely studied, it has been proposed to be secondary to the HDL scavenging of oxidized molecules during LDL oxidation. Some experimental evidences suggest that the cardioprotective properties are commonly attenuated in oxHDL. In this regard, oxHDL particles are less effective in promoting cholesterol transport from cells [19,20] and also induce the expression of proinflammatory molecules in the activated macrophages [21]. However, further investigation is needed to establish the contribution that oxHDL makes during atherosclerotic plaque formation.

Finally, the last review article in this Special Issue focused on the contribution of HDL during fetal development [22]. In the fetal bloodstream, cholesterol is mainly transported by HDL [22]. Fetal HDL particles differ from adult HDL particles [23]. Although the physiological role of fetal HDL remains under debate, their enrichment in APOE suggests that their metabolism might resemble that of adult LDL. In their review, Stadler et al. also emphasized the fetal characteristics of HDL particles and determined the extent to which they were different from those found in maternal serum [22]. It is noteworthy that the alterations in maternal HDL composition and function have been identified in the fetal serum in some pregnancy complications, i.e., gestational diabetes mellitus and preclampsia, thus suggesting a placental transmission pattern. However, the impact of maternally derived HDL changes on fetal development or whether these changes may persist into adulthood deserves future research.

Overall, the articles published in this Special Issue provide evidence that the investigation of HDL subpopulations and their relationship with beneficial HDL functions could be translated into the use of HDL biomarkers in clinical disease and the development of successful HDL-based therapies.

## References

[1] Lorkowski S.W., Smith J.D. (2022). HDL Is Not Dead Yet. Biomedicines.

[2] Bonizzi A., Piuri G., Corsi F., Cazzola R., Mazzucchelli S. (2021). HDL Dysfunctionality: Clinical Relevance of Quality Rather than Quantity. Biomedicines.

[3] Kanbay M., Solak Y., Unal H.U., Kurt Y.G., Gok M., Cetinkaya H., Karaman M., Oguz Y., Eyileten T., Vural A. (2014). Monocyte count/HDL cholesterol ratio and cardiovascular events in patients with chronic kidney disease. Int. Urol. Nephrol..

[4] Lappegard K.T., Kjellmo C.A., Hovland A. (2021). High-Density Lipoprotein Subfractions: Much Ado about Nothing or Clinically Important?. Biomedicines.

[5] Coimbra S., Reis F., Valente M.J., Rocha S., Catarino C., Rocha-Pereira P., Sameiro-Faria M., Bronze-da-Rocha E., Belo L., Santos-Silva A. (2021). Subpopulations of High-Density Lipoprotein: Friends or Foes in Cardiovascular Disease Risk in Chronic Kidney Disease?. Biomedicines.

[6] Vaziri N.D., Navab M., Fogelman A.M. (2010). HDL metabolism and activity in chronic kidney disease. Nat. Rev. Nephrol..

[7] Untersteller K., Meissl S., Trieb M., Emrich I.E., Zawada A.M., Holzer M., Knuplez E., Fliser D., Heine G.H., Marsche G. (2018). HDL functionality and cardiovascular outcome among nondialysis chronic kidney disease patients. J. Lipid Res..

[8] Rysz J., Gluba-Brzozka A., Rysz-Gorzynska M., Franczyk B. (2020). The Role and Function of HDL in Patients with Chronic Kidney Disease and the Risk of Cardiovascular Disease. Int. J. Mol. Sci..

[9] Kuchta A., Strzelecki A., Cwiklinska A., Gruchala M., Zdrojewski Z., Kortas-Stempak B., Wieczorek E., Gliwinska A., Dabkowski K., Jankowski M. (2017). HDL subpopulations containing apoA-I without apoA-II (LpA-I) in patients with angiographically proven coronary artery disease. J. Cardiol..

[10] Prats-Uribe A., Sayols-Baixeras S., Fernandez-Sanles A., Subirana I., Carreras-Torres R., Vilahur G., Civeira F., Marrugat J., Fito M., Hernaez A. (2020). High-density lipoprotein characteristics and coronary artery disease: A Mendelian randomization study. Metabolism.

[11] Didichenko S.A., Navdaev A.V., Cukier A.M., Gille A., Schuetz P., Spycher M.O., Therond P., Chapman M.J., Kontush A., Wright S.D. (2016). Enhanced HDL Functionality in Small HDL Species Produced Upon Remodeling of HDL by Reconstituted HDL, CSL112: Effects on Cholesterol Efflux, Anti-Inflammatory and Antioxidative Activity. Circ. Res..

[12] Pedro-Botet J., Climent E., Benaiges D. (2021). Familial Hypercholesterolemia: Do HDL Play a Role?. Biomedicines.

[13] Canyelles M., Tondo M., Cedo L., Farras M., Escola-Gil J.C., Blanco-Vaca F. (2018). Trimethylamine N-Oxide: A Link among Diet, Gut Microbiota, Gene Regulation of Liver and Intestine Cholesterol Homeostasis and HDL Function. Int. J. Mol. Sci..

[14] Rohatgi A., Westerterp M., von Eckardstein A., Remaley A., Rye K.A. (2021). HDL in the 21st Century: A Multifunctional Roadmap for Future HDL Research. Circulation.

[15] Canyelles M., Garcia-Osuna A., Junza A., Yanes O., Puig N., Ordonez-Llanos J., Sionis A., Sans-Rosello J., Alquezar-Arbe A., Santos D. (2021). The Capacity of APOB-Depleted Plasma in Inducing ATP-Binding Cassette A1/G1-Mediated Macrophage Cholesterol Efflux-But Not Gut Microbial-Derived Metabolites-Is Independently Associated with Mortality in Patients with ST-Segment Elevation Myocardial Infarction. Biomedicines.

[16] Finamore F., Nieddu G., Rocchiccioli S., Spirito R., Guarino A., Formato M., Lepedda A.J. (2021). Apolipoprotein Signature of HDL and LDL from Atherosclerotic Patients in Relation with Carotid Plaque Typology: A Preliminary Report. Biomedicines.

[17] Itabe H., Sawada N., Makiyama T., Obama T. (2021). Structure and Dynamics of Oxidized Lipoproteins In Vivo: Roles of High-Density Lipoprotein. Biomedicines.

[18] Huang Y., DiDonato J.A., Levison B.S., Schmitt D., Li L., Wu Y., Buffa J., Kim T., Gerstenecker G.S., Gu X. (2014). An abundant dysfunctional apolipoprotein A1 in human atheroma. Nat. Med..

[19] Cukier A.M.O., Therond P., Didichenko S.A., Guillas I., Chapman M.J., Wright S.D., Kontush A. (2017). Structure-function relationships in reconstituted HDL: Focus on antioxidative activity and cholesterol efflux capacity. Biochim. Biophys. Acta Mol. Cell Biol. Lipids.

[20] Shao B. (2012). Site-specific oxidation of apolipoprotein A-I impairs cholesterol export by ABCA1, a key cardioprotective function of HDL. Biochim. Biophys. Acta.

[21] Schill R.L., Knaack D.A., Powers H.R., Chen Y., Yang M., Schill D.J., Silverstein R.L., Sahoo D. (2020). Modification of HDL by reactive aldehydes alters select cardioprotective functions of HDL in macrophages. FEBS J..

[22] Stadler J.T., Wadsack C., Marsche G. (2021). Fetal High-Density Lipoproteins: Current Knowledge on Particle Metabolism, Composition and Function in Health and Disease. Biomedicines.

[23] Sreckovic I., Birner-Gruenberger R., Obrist B., Stojakovic T., Scharnagl H., Holzer M., Scholler M., Philipose S., Marsche G., Lang U. (2013). Distinct composition of human fetal HDL attenuates its anti-oxidative capacity. Biochim. Biophys. Acta.

